# Trends and Disparities in Pneumonia-Related Mortality in the U.S. Population: A Nationwide Analysis Using the CDC WONDER Data

**DOI:** 10.7759/cureus.83371

**Published:** 2025-05-02

**Authors:** Chekwube M Obianyo, Nneka Muoghalu, Esther M Adjei, Anthonia N Njoku, Nkiruka L Okoro, Olaide B Sulaiman, Ijeoma M Nwabuokei, Vincent U Barrah

**Affiliations:** 1 Epidemiology and Public Health, Jiann-Ping Hsu College of Public Health, Georgia Southern University, Statesboro, USA; 2 Public Health, School of Tropical Medicine, University of Liverpool, Liverpool, GBR; 3 Internal Medicine, University College Hospital, Ibadan, NGA; 4 Internal Medicine, The Trust Hospital Company Limited, Accra, GHA; 5 Pediatrics and Neonatology, Spine Clinic, UT Southwestern Medical Center, Frisco, USA; 6 Pediatrics, Garki Hospital Abuja, Abuja, NGA; 7 Oncology, Garki Hospital Abuja, Abuja, NGA; 8 Emergency Medicine, Accident and Emergency, Mid Cheshire NHS Trust, Crewe, GBR; 9 Internal Medicine, Kingston Public Hospital, Kingston, JAM; 10 Public Health, Chicago State University, Chicago, USA

**Keywords:** cdc wonder, demographic disparities, icd-10 codes, pneumonia mortality, public health intervention, time trends

## Abstract

Background

Pneumonia remains a significant health concern, particularly affecting older adults, individuals with chronic conditions, and underserved populations. The analysis of pneumonia-related mortality rates based on age groupings and sex affiliation and ethnicity, and geographic region strength is vital because it directs spending distribution and helps design tailored prevention methods for vulnerable patient populations. This study analyzes nationwide trends that can help identify high-risk populations and inform targeted public health interventions.

Methods

Age-adjusted pneumonia mortality rates were computed from CDC WONDER data, which spanned from 1999 until 2023 through direct standardization to the 2000 U.S. standard population and expressed per 100,000 population. The average annual percent change was estimated using log-linear regression. Subgroup disparities were assessed by calculating rate ratios with 95% confidence intervals. Two-sided p-values less than 0.05, based on chi-square tests, were used to evaluate differences across sex, racial/ethnic groups, and yearly trends.

Results

From 1999 to 2023, the age-adjusted pneumonia mortality rate declined from 22.9 to 9.9 per 100,000 population (p < 0.05). Although both sexes experienced reductions (p < 0.05), males consistently had higher mortality (p < 0.05). Black or African American individuals had higher rates than other groups (p < 0.05), despite overall improvements. Mortality increased with age, peaking among those ≥85 years (p < 0.05). Hispanic individuals had consistently lower rates than non-Hispanics(p = 0.05). A spike in mortality occurred in 2020 during the COVID-19 pandemic (p < 0.05), followed by stabilization.

Conclusion

Despite significant declines in pneumonia-related mortality over the past two decades, disparities remain by sex, age, race, and ethnicity. Older adults, males, and Black individuals continue to bear a disproportionate burden. The temporary COVID-19-related increase emphasizes the need for sustained public health focus. Targeted strategies are crucial to address persistent inequities and reduce pneumonia-related deaths further.

## Introduction

Clinical definition and pathophysiology

Pneumonia is defined as an infection of the lung parenchyma, encompassing a spectrum of syndromes caused by diverse pathogens that invade alveolar spaces and lead to consolidation on radiographic imaging [1]. The pathophysiology begins when microorganisms bypass upper airway defenses and enter the lower respiratory tract, triggering an intense inflammatory response characterized by the release of pro-inflammatory cytokines (e.g., IL-1, IL-6, tumour necrosis factor alpha (TNF-α)) and recruitment of neutrophils and macrophages, which results in alveolar fluid accumulation and impaired gas exchange [2]. Clinically, this manifests as cough, fever, dyspnea, tachypnea, and pleuritic chest pain, with potential complications including empyema, sepsis, acute respiratory distress syndrome, and multi-organ failure [2]. Among bacterial causes, *Streptococcus pneumoniae *and *Haemophilus influenzae *remain the most prevalent pathogens, while viruses, particularly influenza viruses and SARS-CoV-2, have been increasingly recognized as major contributors to severe pneumonia, especially during seasonal epidemics and the COVID-19 pandemic [3].

Global disease burden

Pneumonia is the leading infectious cause of death in children under five, claiming an estimated 740,180 lives globally in 2019 (14% of under-5 mortality), and remains a top cause of death across all age groups worldwide [4]. In hospitalized adults, case fatality can be as high as 22-23% within 30 days of admission, underscoring the severity of severe pneumonia and its strain on healthcare systems [5]. Despite being largely preventable and treatable, pneumonia causes over 2.5 million deaths annually, highlighting critical gaps in vaccination coverage, nutrition, and environmental interventions in low- and middle-income settings [6].

U.S. epidemiology and disparities

In the United States, approximately 1.5 million adults are diagnosed with pneumonia each year, leading to around 1 million hospitalizations and 50,000 deaths, with older adults (≥65 years) bearing the greatest burden [7]. National data show that in 2020, pneumonia accounted for over 53,000 deaths and 2.6 million emergency department visits, making it one of the top ten causes of mortality and a leading driver of healthcare utilization [8]. Geographic analyses reveal that the South census region, including states such as Mississippi and Alabama, consistently reports higher age-adjusted pneumonia mortality rates compared with other regions [8]. Moreover, nonmetropolitan (rural) counties exhibit higher age-adjusted pneumonia mortality than metropolitan areas, reflecting disparities in healthcare access, socioeconomic conditions, and public health infrastructure [9].

Objective

The primary objective of this study is to investigate national trends and disparities in pneumonia-related mortality in the United States from 1999 through 2023, with a focus on demographic (age, sex, race/ethnicity) and geographic (state, census region, and urban-rural classification) variations using CDC WONDER data [10]. This research seeks to provide valuable insights that can inform public health policies, enhance preventive strategies, and improve clinical management of pneumonia by identifying key factors contributing to mortality rates. Ultimately, these findings will contribute to ongoing efforts to reduce the burden of pneumonia and improve overall population health outcomes.

## Materials and methods

Study design

This study employed a retrospective, cross-sectional design to analyze pneumonia-related mortality trends in the United States over the period from 1999 to 2023. The design enabled assessment of demographic (age, sex, race/ethnicity) and temporal patterns without intervention or individual-level exposure assignment. We ensured transparent reporting of objectives, methods, and findings by adhering to Strengthening the Reporting of Observational Studies in Epidemiology (STROBE) guidelines for observational research.

Data source

Mortality data for years 1999-2023 were retrieved from the CDC WONDER system, which aggregates National Vital Statistics System death‐certificate records [10]. WONDER provides public access to detailed cause-of-death information coded by ICD-10, enabling robust epidemiologic analyses [10]. Pneumonia-related deaths were identified using ICD-10 codes J12-J18, which encompass viral (J12), bacterial, and unspecified pneumonia diagnoses.

Inclusion and exclusion criteria

All recorded deaths in the WONDER database from 1999 to 2023 with ICD-10 codes J12-J18 listed as primary or contributing causes were included. Inclusion spanned all ages, sexes, and race/ethnicity groups to maximize representativeness of U.S. mortality patterns. No exclusion criteria were applied, ensuring comprehensive coverage of pneumonia-related mortality over the study period.

Data extraction and variables

Data extraction was conducted using the CDC WONDER online query system, which facilitates filtering based on demographic and geographic characteristics. The dataset included key variables such as age, sex, race/ethnicity, state of residence, and year of death. Age groups were categorized as <1 year, 1-4 years, 5-14 years, 15-24 years, 25-34 years, 35-44 years, 45-54 years, 55-64 years, 65-74 years, 75-84 years, and 85+ years. Sex was classified as male or female. Racial/ethnic categories included American Indian or Alaska Native, Asian, Black or African American, Native Hawaiian or Other Pacific Islander, White, and individuals identifying with more than one race. Additionally, Hispanic ethnicity was classified as Hispanic or Latino, Not Hispanic or Latino, or Not Stated. The primary outcome variable was pneumonia-related mortality, assessed using ICD-10 codes J12-J18. The study analyzed variations in mortality rates based on age group, racial/ethnic disparities, and geographic distribution across U.S. states.

Data analysis

Descriptive statistics (frequencies, percentages) characterized demographic distributions and raw mortality counts. Age-adjusted mortality rates per 100,000 population were computed using the direct standardization method with the 2000 U.S. standard population, eliminating confounding by age distribution differences across groups. Comparative analyses between subgroups (sex, race/ethnicity) employed chi-square tests for proportions and independent-samples t-tests or one-way ANOVA, as appropriate. Temporal trends in log-transformed age-adjusted rates were assessed via linear regression to estimate average annual percent change (APC), reflecting constant yearly percentage shifts. All statistical procedures were performed in SPSS version 26.0 (IBM Corp., Armonk, USA), with two-sided p < 0.05 indicating statistical significance.

Ethical considerations

This study used publicly available, de-identified mortality data from CDC WONDER, which implements privacy safeguards (e.g., small cell suppression) to prevent disclosure of personally identifiable information [10]. Analysis of such secondary, non-identifiable data is exempt from human subjects review under federal regulations and institutional guidance on secondary data analysis. All procedures adhered to CDC WONDER data use policies as described in the system’s help documentation [10].

## Results

The results highlight pneumonia-related mortality trends in the U.S., emphasizing demographic disparities, geographic variations, and seasonal patterns. Between 1999 and 2023, deaths declined from 62,065 to 41,210, with the age-adjusted mortality rate dropping from 22.9 to 9.9 per 100,000. Narrowing confidence intervals (1999: 22.7-23.1; 2023: 9.7-9.9) indicates greater estimate precision. Statistical tests comparing mortality rates across sex, race/ethnicity, and age groups all yielded p < 0.05, suggesting that the observed differences between males and females, among racial/ethnic groups, and across age strata are unlikely to be due to chance (Table 1).

**Table 1:** Patterns of underlying causes of death (age-adjusted mortality rate) due to pneumonia (1999-2023) Values outside parentheses indicate age-adjusted mortality rates per 100,000 population; values in parentheses represent 95% confidence intervals (CI).

Characteristics	1999	2000	2001	2002	2003	2004	2005	2006	2007	2008	2009	2010	2011	2012	2013	2014	2015	2016	2017	2018	2019	2020	2021	2022	2023	P value
Deaths, n	62,065	63,548	61,777	64,954	63,371	58,564	61,189	55,477	52,306	54,562	50,774	49,597	52,294	49,530	53,282	50,622	51,811	48,632	49,157	47,956	43,881	47,601	41,309	41,108	41,210	
Population, n	279040168	281421906	284968955	287625193	290107933	292805298	295516599	298379912	301231207	304093966	306771529	308745538	311591917	313914040	316128839	318857056	321418820	323127513	325719178	327167434	328239523	329484123	331893745	333287557	334914895	
Age-Adjusted Rate (95% CI)	22.9 (22.7 - 23.1)	23.1 (22.9 - 23.3)	22.1 (21.9 - 22.2)	22.9 (22.7 - 23.1)	22.0 (21.8 - 22.1)	20.0 (19.8 - 20.2)	20.4 (20.3 - 20.6)	18.1 (18.0 - 18.3)	16.7 (16.6 - 16.9)	17.1 (16.9 - 17.2)	15.6 (15.5 - 15.7)	14.9 (14.8 - 15.1)	15.3 (15.2 - 15.4)	14.2 (14.0 - 14.3)	14.8 (14.7 - 15.0)	13.8 (13.7 - 13.9)	13.8 (13.7 - 14.0)	12.8 (12.6 - 12.9)	12.6 (12.5 - 12.7)	12.0 (11.9 - 12.1)	10.8 (10.7 - 10.9)	11.6 (11.5 - 11.7)	10.4 (10.3 - 10.5)	9.8 (9.7 - 9.9)	9.9 (9.8 - 10.0)	
Based on sex																										
Female	20.0 (19.7 - 20.2)	20.1 (19.9 - 20.3)	19.2 (19.0 - 19.4)	20.0 (19.8 - 20.2)	19.2 (19.0 - 19.4)	17.4 (17.2 - 17.6)	18.0 (17.8 - 18.2)	15.8 (15.6 - 16.0)	14.6 (14.4 - 14.8)	15.0 (14.8 - 15.2)	13.6 (13.5 - 13.8)	12.9 (12.8 - 13.1)	13.2 (13.1 - 13.4)	12.2 (12.1 - 12.4)	13.0 (12.8 - 13.2)	12.1 (11.9 - 12.2)	12.1 (11.9 - 12.2)	11.1 (11.0 - 11.3)	11.1 (10.9 - 11.2)	10.4 (10.3 - 10.6)	9.4 (9.2 - 9.5)	9.6 (9.5 - 9.8)	8.5 (8.4 - 8.6)	8.3 (8.1 - 8.4)	8.4 (8.2 - 8.5)	<0.05
Male	27.9 (27.5 - 28.2)	28.2 (27.9 - 28.5)	26.8 (26.5 - 27.1)	27.6 (27.3 - 28.0)	26.3 (26.0 - 26.6)	24.2 (23.9 - 24.5)	24.3 (24.0 - 24.6)	21.8 (21.5 - 22.1)	20.0 (19.8 - 20.3)	20.1 (19.9 - 20.4)	18.6 (18.3 - 18.8)	18.0 (17.8 - 18.3)	18.3 (18.1 - 18.6)	17.0 (16.7 - 17.2)	17.5 (17.2 - 17.7)	16.4 (16.2 - 16.6)	16.3 (16.1 - 16.5)	15.0 (14.8 - 15.2)	14.7 (14.5 - 14.9)	14.2 (14.0 - 14.4)	12.8 (12.6 - 13.0)	14.1 (14.0 - 14.3)	12.9 (12.7 - 13.0)	12.0 (11.9 - 12.2)	11.8 (11.7 - 12.0)	
Based on race/ethnicity																										
American Indian or Alaska Native	27.7 (24.4 - 31.0)	22.0 (19.3 - 24.7)	23.8 (21.0 - 26.6)	22.3 (19.6 - 25.1)	25.9 (23.1 - 28.8)	19.6 (17.2 - 22.1)	22.7 (20.2 - 25.3)	16.2 (14.1 - 18.3)	15.6 (13.6 - 17.7)	18.8 (16.6 - 20.9)	15.6 (13.8 - 17.5)	15.8 (13.9 - 17.7)	14.6 (12.9 - 16.3)	12.8 (11.2 - 14.3)	14.0 (12.4 - 15.6)	13.8 (12.2 - 15.3)	11.2 (9.8 - 12.5)	11.9 (10.6 - 13.2)	11.9 (10.7 - 13.2)	10.8 (9.7 - 12.0)	9.4 (8.3 - 10.4)	11.0 (9.9 - 12.1)	10.0 (8.9 - 11.1)	9.3 (8.3 - 10.3)	9.0 (8.0 - 10.0)	<0.05
Asian or Pacific Islander	15.9 (14.8 - 17.0)	19.5 (18.3 - 20.7)	19.2 (18.1 - 20.4)	18.1 (17.1 - 19.2)	18.0 (16.9 - 19.0)	16.9 (15.9 - 17.8)	16.5 (15.6 - 17.4)	15.9 (15.1 - 16.8)	14.9 (14.1 - 15.7)	15.4 (14.6 - 16.2)	14.3 (13.5 - 15.0)	14.3 (13.6 - 15.1)	13.9 (13.2 - 14.6)	13.9 (13.2 - 14.5)	14.3 (13.6 - 14.9)	12.3 (11.7 - 12.8)	13.2 (12.7 - 13.8)	12.3 (11.7 - 12.8)	11.8 (11.3 - 12.3)	11.0 (10.6 - 11.5)	8.6 (8.2 - 9.1)	9.4 (9.0 - 9.8)	8.0 (7.6 - 8.4)	7.7 (7.3 - 8.0)	8.2 (7.8 - 8.6)	
Black or African American	25.1 (24.4 - 25.8)	25.3 (24.6 - 25.9)	24.3 (23.7 - 25.0)	24.5 (23.9 - 25.2)	23.6 (23.0 - 24.3)	22.9 (22.3 - 23.5)	22.4 (21.8 - 22.9)	20.3 (19.7 - 20.8)	19.1 (18.5 - 19.6)	19.2 (18.7 - 19.8)	17.2 (16.7 - 17.7)	16.7 (16.2 - 17.2)	16.5 (16.0 - 16.9)	15.6 (15.1 - 16.0)	16.0 (15.5 - 16.4)	15.1 (14.7 - 15.5)	15.1 (14.6 - 15.5)	14.4 (14.0 - 14.8)	13.7 (13.3 - 14.1)	13.5 (13.2 - 13.9)	12.1 (11.8 - 12.5)	14.9 (14.5 - 15.3)	12.9 (12.5 - 13.2)	11.5 (11.2 - 11.8)	11.4 (11.0 - 11.7)	
White	22.7 (22.5 - 22.9)	22.9 (22.7 - 23.1)	21.8 (21.6 - 22.0)	22.8 (22.6 - 23.0)	21.8 (21.6 - 21.9)	19.7 (19.6 - 19.9)	20.3 (20.1 - 20.4)	17.9 (17.8 - 18.1)	16.5 (16.3 - 16.6)	16.8 (16.6 - 16.9)	15.4 (15.3 - 15.5)	14.7 (14.6 - 14.9)	15.2 (15.0 - 15.3)	14.0 (13.8 - 14.1)	14.7 (14.6 - 14.8)	13.7 (13.6 - 13.8)	13.7 (13.6 - 13.8)	12.5 (12.4 - 12.7)	12.5 (12.3 - 12.6)	11.9 (11.8 - 12.0)	10.8 (10.7 - 10.9)	11.2 (11.1 - 11.3)	10.2 (10.1 - 10.3)	9.8 (9.7 - 9.9)	9.8 (9.7 - 9.9)	
Native Hawaiian or Other Pacific Islander	-	-	-	-	-	-	-	-	-	-	-	-	-	-	-		-	-	-	-	-	-	7.9 (6.7 - 9.1)	5.9 (4.9 - 7.0)	10.4 (9.0 - 11.6)	
More than One Race	-	-	-	-	-	-	-	-	-	-	-	-	-	-	-	-	-	-	-	-	-	-	4.1 (4.0 - 4.5)	3.5 (3.4 - 3.9)	4.7 (4.6 - 5.1)	
Based on age groups																										
< 1 year	8.1 (7.2 - 9.0)	7.4 (6.5 - 8.2)	7.3 (6.4 - 8.1)	6.5 (5.7 - 7.3)	7.3 (6.5 - 8.2)	6.4 (5.6 - 7.1)	6.1 (5.3 - 6.9)	6.1 (5.3 - 6.9)	5.0 (4.2 - 5.8)	5.1 (4.3 - 5.9)	5.2 (4.4 - 6.0)	4.5 (3.9 - 5.2)	4.6 (3.9 - 5.2)	3.7 (3.1 - 4.3)	4.0 (3.4 - 4.6)	4.0 (3.3 - 4.6)	3.9 (3.3 - 4.5)	3.9 (4.5 - 3.2)	3.7 (4.3 - 3.1)	4.1 (4.7 - 3.4)	3.5 (4.1 - 2.9)	3.0 (3.5 - 2.4)	3.4 (4.0 - 2.8)	3.9 (4.5 - 3.2)	3.6 (4.2 - 3.0)	<0.05
1-4 years	0.8 (0.6 - 0.9)	0.6 (0.5 - 0.7)	0.7 (0.6 - 0.8)	0.7 (0.5 - 0.8)	0.7 (0.5 - 0.8)	0.6 (0.5 - 0.8)	0.6 (0.4 - 0.8)	0.7 (0.5 - 0.9)	0.6 (0.4 - 0.8)	0.7 (0.5 - 0.9)	0.6 (0.4 - 0.8)	0.5 (0.4 - 0.6)	0.6 (0.4 - 0.7)	0.5 (0.4 - 0.6)	0.4 (0.3 - 0.6)	0.4 (0.3 - 0.6)	0.4 (0.3 - 0.5)	0.5 (0.6 - 0.4)	0.4 (0.6 - 0.3)	0.4 (0.6 - 0.3)	0.4 (0.5 - 0.3)	0.2 (0.3 - 0.2)	0.3 (0.4 - 0.2)	0.5 (0.6 - 0.4)	0.7 (0.8 - 0.5)	
5-14 years	0.2 (0.2 - 0.2)	0.2 (0.1 - 0.2)	0.2 (0.2 - 0.2)	0.2 (0.2 - 0.2)	0.2 (0.2 - 0.3)	0.2 (0.1 - 0.2)	0.2 (0.1 - 0.3)	0.1 (0.0 - 0.2)	0.2 (0.1 - 0.3)	0.1 (0.0 - 0.2)	0.2 (0.1 - 0.3)	0.1 (0.1 - 0.2)	0.2 (0.2 - 0.3)	0.1 (0.1 - 0.2)	0.2 (0.1 - 0.2)	0.1 (0.1 - 0.1)	0.1 (0.1 - 0.2)	0.1 (0.2 - 0.1)	0.1 (0.2 - 0.1)	0.1 (0.2 - 0.1)	0.1 (0.2 - 0.1)	0.1 (0.1 - 0.1)	0.1 (0.1 - 0.1)	0.1 (0.1 - 0.1)	0.2 (0.2 - 0.1)	
15-24 years	0.4 (0.4 - 0.5)	0.4 (0.4 - 0.5)	0.4 (0.4 - 0.5)	0.4 (0.3 - 0.5)	0.5 (0.4 - 0.5)	0.4 (0.4 - 0.5)	0.4 (0.3 - 0.5)	0.4 (0.3 - 0.5)	0.4 (0.3 - 0.5)	0.4 (0.3 - 0.5)	0.5 (0.4 - 0.6)	0.3 (0.3 - 0.4)	0.4 (0.3 - 0.4)	0.3 (0.3 - 0.4)	0.3 (0.3 - 0.4)	0.3 (0.3 - 0.4)	0.4 (0.3 - 0.4)	0.3 (0.4 - 0.3)	0.3 (0.4 - 0.3)	0.3 (0.4 - 0.3)	0.3 (0.3 - 0.2)	0.3 (0.3 - 0.2)	0.3 (0.3 - 0.2)	0.2 (0.3 - 0.2)	0.3 (0.3 - 0.2)	
25-34 years	0.8 (0.7 - 0.9)	0.9 (0.8 - 1.0)	0.8 (0.7 - 0.9)	0.9 (0.8 - 1.0)	0.9 (0.8 - 1.0)	0.8 (0.7 - 0.8)	0.9 (0.8 - 1.0)	0.8 (0.7 - 0.9)	0.8 (0.7 - 0.9)	0.9 (0.8 - 1.0)	1.2 (1.1 - 1.3)	0.8 (0.7 - 0.9)	1.0 (0.9 - 1.1)	0.8 (0.7 - 0.9)	0.9 (0.8 - 1.0)	0.9 (0.8 - 1.0)	0.8 (0.7 - 0.9)	0.8 (0.9 - 0.7)	0.8 (0.9 - 0.7)	0.7 (0.8 - 0.7)	0.7 (0.8 - 0.7)	0.9 (1.0 - 0.8)	0.9 (0.9 - 0.8)	0.8 (0.9 - 0.7)	0.8 (0.9 - 0.8)	
35-44 years	2.3 (2.2 - 2.5)	2.3 (2.2 - 2.5)	2.2 (2.0 - 2.3)	2.2 (2.0 - 2.3)	2.2 (2.1 - 2.3)	2.0 (1.9 - 2.1)	2.1 (1.9 - 2.3)	1.9 (1.7 - 2.1)	1.8 (1.6 - 2.0)	2.0 (1.8 - 2.2)	2.3 (2.1 - 2.5)	1.8 (1.6 - 1.9)	1.9 (1.7 - 2.0)	1.6 (1.5 - 1.7)	1.8 (1.7 - 1.9)	1.9 (1.8 - 2.0)	1.6 (1.5 - 1.7)	1.8 (1.9 - 1.7)	1.6 (1.8 - 1.5)	1.7 (1.8 - 1.5)	1.7 (1.8 - 1.5)	2.1 (2.3 - 2.0)	1.9 (2.0 - 1.8)	1.8 (1.9 - 1.6)	1.8 (1.9 - 1.7)	
45-54 years	4.6 (4.3 - 4.8)	4.6 (4.4 - 4.8)	4.5 (4.3 - 4.7)	4.8 (4.5 - 5.0)	5.1 (4.9 - 5.3)	4.5 (4.3 - 4.7)	5.0 (4.8 - 5.2)	4.6 (4.4 - 4.8)	4.3 (4.1 - 4.5)	4.9 (4.7 - 5.1)	5.0 (4.8 - 5.2)	4.1 (3.9 - 4.2)	4.6 (4.4 - 4.8)	4.0 (3.8 - 4.2)	4.5 (4.3 - 4.7)	4.9 (4.7 - 5.2)	4.4 (4.2 - 4.6)	4.2 (4.4 - 4.0)	4.2 (4.4 - 4.0)	4.2 (4.4 - 4.0)	4.0 (4.2 - 3.8)	4.9 (5.2 - 4.7)	4.3 (4.5 - 4.1)	3.9 (4.1 - 3.8)	3.7 (3.9 - 3.5)	
55-64 years	10.7 (10.3 - 11.2)	11.5 (11.1 - 11.9)	10.7 (10.3 - 11.1)	11.1 (10.7 - 11.5)	10.9 (10.5 - 11.3)	10.6 (10.2 - 10.9)	11.0 (10.6 - 11.4)	9.7 (9.3 - 10.1)	9.4 (9.0 - 9.8)	10.6 (10.2 - 11.0)	10.4 (10.0 - 10.8)	9.7 (9.4 - 10.0)	10.5 (10.1 - 10.8)	10.0 (9.6 - 10.3)	11.2 (10.9 - 11.5)	11.2 (10.9 - 11.5)	10.4 (10.1 - 10.7)	10.8 (11.1 - 10.5)	10.6 (10.9 - 10.3)	10.8 (11.1 - 10.5)	10.1 (10.4 - 9.8)	12.4 (12.7 - 12.1)	11.1 (11.4 - 10.8)	10.2 (10.5 - 9.9)	9.7 (10.0 - 9.4)	
65-74 years	36.4 (35.5 - 37.2)	38.0 (37.1 - 38.9)	36.1 (35.2 - 36.9)	36.9 (36.1 - 37.8)	35.9 (35.1 - 36.8)	33.7 (32.8 - 34.5)	34.4 (33.6 - 35.2)	31.1 (30.3 - 31.9)	28.0 (27.2 - 28.8)	29.9 (29.1 - 30.7)	28.4 (27.6 - 29.2)	27.8 (27.1 - 28.5)	28.2 (27.5 - 28.9)	25.7 (25.0 - 26.3)	27.9 (27.3 - 28.6)	27.4 (26.8 - 28.1)	27.3 (26.7 - 27.9)	26.8 (27.4 - 26.2)	26.5 (27.1 - 25.9)	26.1 (26.7 - 25.6)	23.8 (24.3 - 23.3)	28.4 (29.0 - 27.9)	24.9 (25.4 - 24.3)	24.4 (25.0 - 23.9)	23.7 (24.2 - 23.2)	
75-84 years	153.3 (151.1 - 155.5)	156.4 (154.2 - 158.6)	147.9 (145.7 - 150.0)	155.2 (153.0 - 157.3)	147.2 (145.1 - 149.3)	136.8 (134.8 - 138.8)	138.3 (136.5 - 140.1)	125.5 (123.7 - 127.3)	112.9 (111.1 - 114.7)	115.5 (113.7 - 117.3)	105.4 (103.6 - 107.2)	102 (100.3 - 103.8)	102.4 (100.7 - 104.2)	96.7 (95.0 - 98.3)	98.6 (96.9 - 100.3)	91.2 (89.6 - 92.8)	92.9 (91.3 - 94.5)	85.1 (86.6 - 83.6)	83.9 (85.4 - 82.4)	78.1 (79.5 - 76.7)	69.9 (71.2 - 68.6)	76.5 (77.8 - 75.2)	68.9 (70.2 - 67.6)	64.5 (65.7 - 63.3)	63.3 (64.4 - 62.1)	
85+ years	730.6 (722.4 - 738.8)	723.9 (715.8 - 732.0)	697.9 (690.0 - 705.7)	723.1 (715.1 - 731.1)	685.0 (677.3 - 692.7)	610.6 (603.4 - 617.8)	624.0 (615.8 - 632.2)	539.4 (531.2 - 547.6)	503.9 (495.7 - 512.1)	496.6 (488.4 - 504.8)	430.0 (421.8 - 438.2)	425.1 (419.6 - 430.5)	430.9 (425.5 - 436.2)	399.9 (394.8 - 405.1)	414.7 (409.5 - 419.8)	368.1 (363.3 - 372.9)	378.1 (373.3 - 382.9)	330.1 (334.6 - 325.7)	329.8 (334.2 - 325.3)	308.9 (313.1 - 304.6)	270.7 (274.7 - 266.8)	254.1 (257.9 - 250.3)	229.6 (233.5 - 225.8)	217.9 (221.5 - 214.3)	229.0 (232.8 - 225.2)	
Based on Hispanic race																										
Hispanic or Latino	18.5 (17.7 - 19.3)	20.3 (19.5 - 21.2)	20.6 (19.8 - 21.4)	20.2 (19.4 - 21.0)	19.2 (18.5 - 20.0)	18.4 (17.7 - 19.2)	18.2 (17.5 - 18.9)	16.6 (15.9 - 17.2)	14.6 (14.0 - 15.2)	15.6 (15.1 - 16.2)	14.2 (13.7 - 14.7)	13.4 (12.9 - 13.9)	13.3 (12.8 - 13.7)	11.8 (11.4 - 12.2)	12.4 (12.0 - 12.9)	11.6 (11.2 - 12.0)	10.8 (10.4 - 11.2)	10.3 (10.0 - 10.7)	10.0 (9.7 - 10.4)	9.8 (9.5 - 10.1)	8.6 (8.3 - 8.9)	10.7 (10.4 - 11.1)	9.3 (9.0-9.8)	8.9 (8.7-9.2)	8.9 (8.7-9.3)	0.05
Not Hispanic or Latino	23.0 (22.8 - 23.2)	23.1 (22.9 - 23.3)	22.1 (21.9 - 22.3)	23.0 (22.8 - 23.2)	22.1 (21.9 - 22.2)	20.0 (19.9 - 20.2)	20.5 (20.4 - 20.7)	18.2 (18.0 - 18.4)	16.8 (16.7 - 17.0)	17.2 (17.0 - 17.3)	15.6 (15.5 - 15.8)	15.1 (14.9 - 15.2)	15.4 (15.3 - 15.6)	14.3 (14.1 - 14.4)	15.0 (14.9 - 15.2)	14.0 (13.8 - 14.1)	14.1 (13.9 - 14.2)	13.0 (12.8 - 13.1)	12.9 (12.7 - 13.0)	12.2 (12.1 - 12.4)	11.1 (11.0 - 11.2)	11.6 (11.5 - 11.7)	10.6 (10.0-10.9)	10.1 (9.7-10.8)	10.1 (9.7-10.8)	

Based on gender

**Figure 1:**
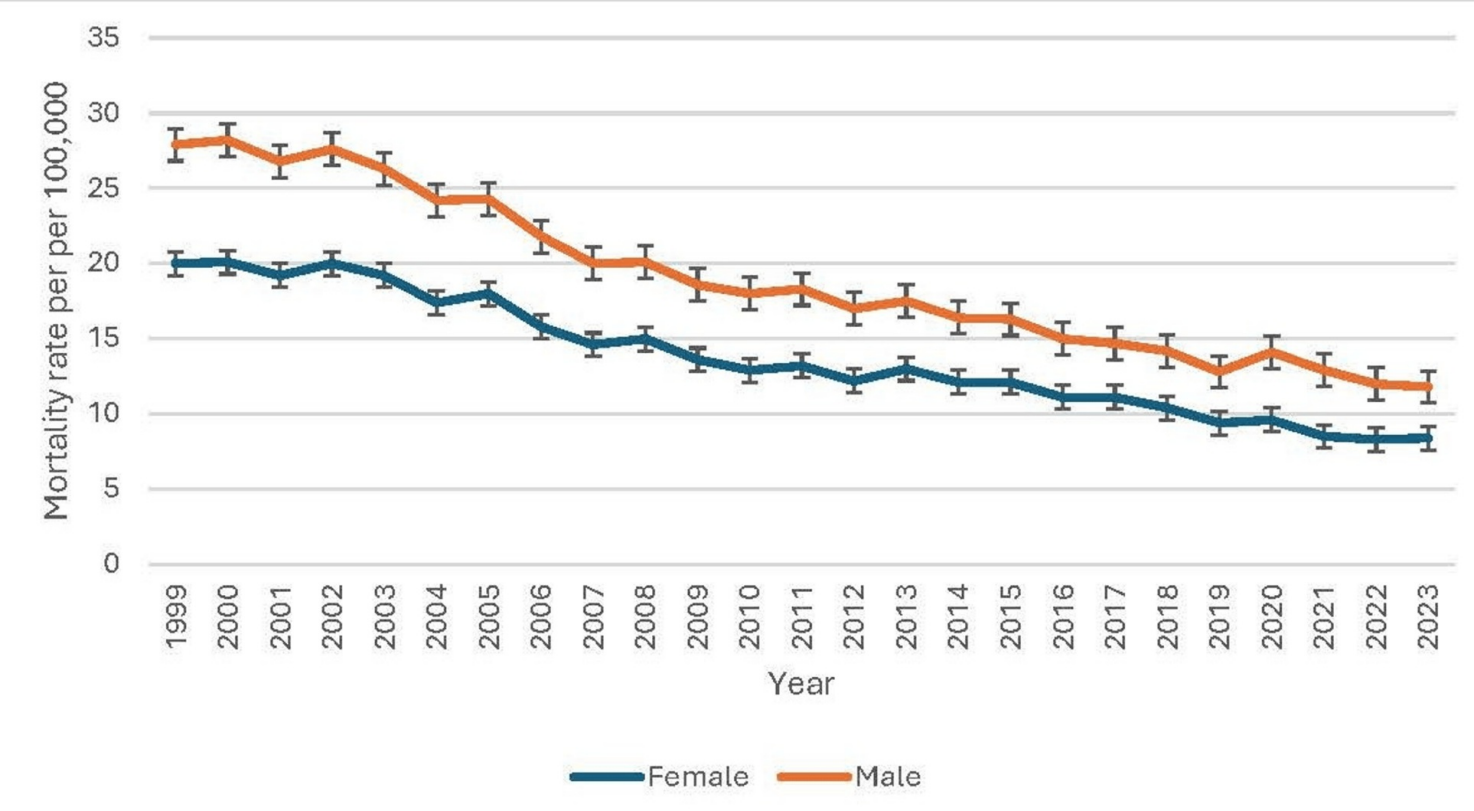
Patterns of underlying causes of death due to pneumonia based on gender

Mortality trends reveal a steady decline in pneumonia‐related deaths among both females and males. From 1999 to 2023, female deaths fell from 34,949 to 19,837, and the age‐adjusted mortality rate dropped from 20.0 to 8.4 per 100,000 (95% CI: 19.7-20.2 to 8.2-8.5), reflecting improvements in healthcare access and targeted preventive efforts for women. Male deaths likewise declined, from 27,116 in 1999 to 23,560 in 2023, and the age‐adjusted rate decreased from 27.9 to 11.8 per 100,000 (95% CI: 27.5-28.2 to 11.7-12.0), although males consistently exhibited higher rates than females. This persistent gender gap may stem from higher smoking prevalence, greater burden of chronic lung disease, and occupational exposures among men, and possible biological differences in immune response [7]. As illustrated in Figure 1, both genders show a consistent downward trend in age‐adjusted mortality rates due to pneumonia, with males maintaining higher rates than females throughout the observed period.

Based on race

**Figure 2:**
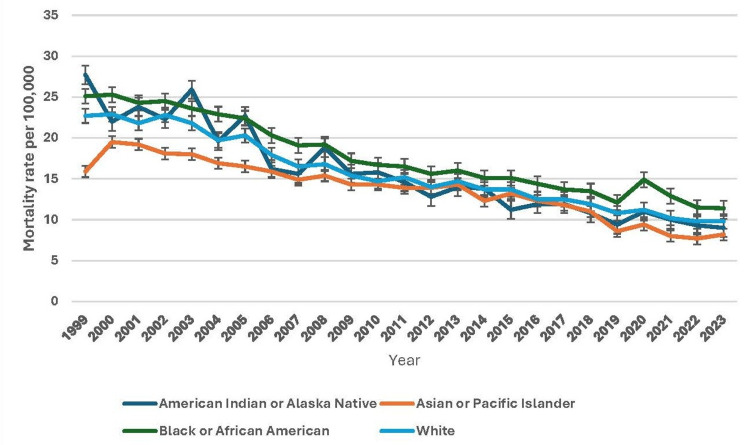
Patterns of underlying causes of death due to pneumonia based on race.

From 1999 to 2023, pneumonia-related mortality trends varied significantly across racial groups. The American Indian or Alaska Native population showed fluctuations, with an age-adjusted mortality rate of 27.7 per 100,000 in 1999 (95% CI: 24.4-31.0), declining to 9.0 in 2023 (95% CI: 8.0-10.0). The Asian or Pacific Islander group experienced a steady decrease from 15.9 in 1999 (95% CI: 14.8-17.0) to 8.2 in 2023 (95% CI: 7.8-8.6). The Black or African American population consistently had higher mortality rates but showed an overall decline from 25.1 in 1999 (95% CI: 24.4-25.8) to 11.4 in 2023 (95% CI: 11.0-11.7). Similarly, the White population saw a steady decrease from 22.7 in 1999 (95% CI: 22.5-22.9) to 9.8 in 2023 (95% CI: 9.7-9.9). More recent data includes the Native Hawaiian or Other Pacific Islander and Multiracial groups. In 2021, the Native Hawaiian or Other Pacific Islander mortality rate was 7.9 (95% CI: 5.9-10.4), rising slightly to 9.1 in 2023 (95% CI: 7.0-11.6). The Multiracial group had the lowest mortality rates, from 4.1 in 2021 (95% CI: 3.5-4.7) to 4.0 in 2023 (95% CI: 3.4-4.6). Despite overall declines, racial disparities persist, with Black or African American individuals continuing to experience higher mortality rates. Figure 2 illustrates the patterns of underlying causes of death due to pneumonia based on race.

Based on age groups

**Figure 3:**
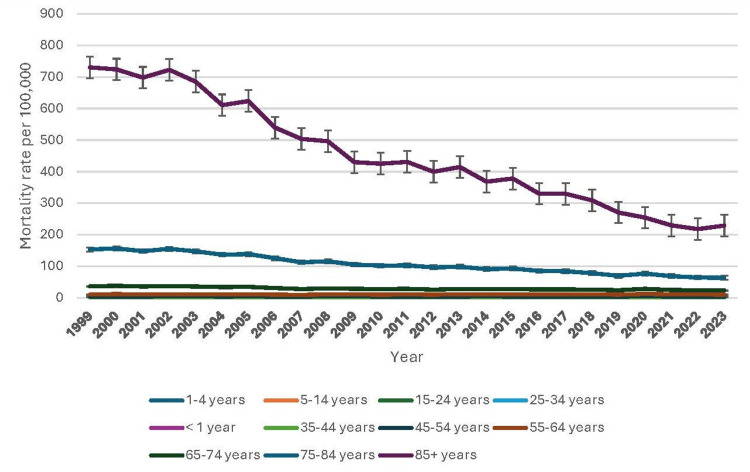
Patterns of underlying causes of death due to pneumonia based on age groups.

Pneumonia-related mortality rates varied significantly across age groups, with older populations experiencing the highest crude death rates per 100,000 population. Among infants (<1 year), mortality declined from 7.2 (95% CI: 7.2-9.0) in 1999 to 3.9 (95% CI: 3.9-5.2) in 2010, reflecting advancements in neonatal care. In children (1-14 years), rates remained low, declining slightly in 1-4-year-olds from 0.6 (95% CI: 0.6-0.9) in 1999 to 0.4 (95% CI: 0.4-0.6) in 2010, while fluctuating between 0.1-0.3 per 100,000 in 5-14-year-olds. Young adults (15-34 years) exhibited low mortality, with stable rates in the 15-24 group (0.4 per 100,000) and a slight increase in the 25-34 group from 0.7 (95% CI: 0.7-0.9) in 1999 to 1.1 (95% CI: 1.1-1.3) in 2009. Among middle-aged adults (35-54 years), mortality declined slightly, with rates in the 35-44 group falling from 2.2 (95% CI: 2.2-2.5) in 1999 to 1.6 (95% CI: 1.6-1.9) in 2010, while the 45-54 group saw a reduction from 4.3 (95% CI: 4.3-4.8) to 3.9 (95% CI: 3.9-4.2). Older adults (55-74 years) experienced an upward trend, peaking in 55-64-year-olds at 10.3 (95% CI: 10.3-11.2) in 1999 before declining slightly. The highest mortality was among those 75+, with rates in the 75-84 group dropping from 151.1 (95% CI: 151.1-155.5) in 1999 to 103.6 (95% CI: 103.6-107.2) in 2009, while those 85+ remained elevated at 424.4 (95% CI: 424.4-435.5) in 2009 (Figure 3). Overall, the mortality rates followed an expected pattern, increasing with age. A declining trend was observed in infant and child mortality, while older age groups exhibited sustained high mortality rates. Figure 3 indicates patterns of underlying causes of death due to pneumonia based on age groups.

Based on Hispanic origin

**Figure 4:**
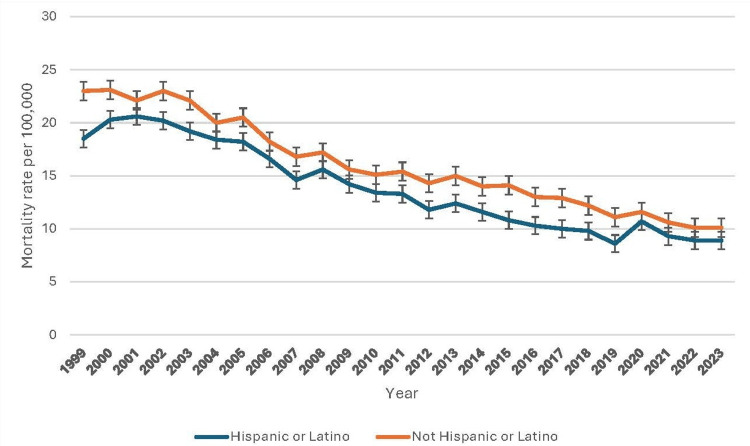
Patterns of underlying causes of death due to pneumonia based on Hispanic race.

From 1999 to 2023, pneumonia-related mortality rates exhibited notable trends among Hispanic and non-Hispanic populations. In 1999, the crude mortality rate for Hispanics was 6.5 per 100,000 (95% CI: 6.2-6.8), with an age-adjusted rate of 18.5 (95% CI: 17.7-19.3). Non-Hispanic individuals had higher rates, with crude and age-adjusted rates of 24.3 (95% CI: 24.1-24.5) and 23.0 (95% CI: 22.8-23.2), respectively. By 2010, mortality declined in both groups, with Hispanic rates at 5.8 (95% CI: 5.6-6.0) crude and 13.4 (95% CI: 12.9-13.9) age-adjusted, while non-Hispanic rates fell to 18.0 (95% CI: 17.9-18.2) crude and 15.1 (95% CI: 14.9-15.2) age-adjusted. A further decline occurred until 2019, but 2020 saw a sharp increase, likely due to COVID-19. Rates stabilized post-pandemic, though disparities persisted, with non-Hispanic individuals consistently exhibiting higher mortality. Figure 4 indicates patterns of underlying causes of death due to pneumonia based on Hispanic race.

## Discussion

These descriptive findings underscore notable progress in reducing pneumonia-related mortality over the past two decades. The overall decline likely reflects successful public health measures, such as expanded pneumococcal and influenza vaccination, improvements in antibiotic stewardship, better access to healthcare services, and advances in clinical management, including earlier diagnosis and more effective supportive care [7]. However, the persistence of significant disparities by sex, race/ethnicity, and age group highlights the need for tailored interventions: clinicians should maintain vigilance in high-risk populations, and policymakers must prioritize resource allocation to underserved communities to further close these gaps.

Pneumonia-related mortality rates clearly increase with advancing age, with the highest burden observed among older adults [11]. The crude and age-adjusted mortality rates increase significantly in individuals aged 65 and older compared to younger age groups. This trend underscores the heightened vulnerability of elderly populations to respiratory infections, likely due to immunosenescence, comorbidities, and prolonged hospital stays [12]. In contrast, younger age groups, particularly those under 45, exhibit considerably lower pneumonia-related mortality rates [13]. However, periodic surges in pneumonia-related deaths among infants and young children highlight the need for enhanced preventive measures such as vaccinations and early medical interventions.

Bondarchuk et al. (2025) reported that pneumonia-related mortality rates were higher in males than females, with an age-adjusted mortality rate ratio (AAMRR) of 1.35 (95% CI: 1.34-1.35) [14]. Similarly, Corica et al. (2022) found consistent gender-based disparities in pneumonia mortality, with males experiencing a higher burden across all analyzed years. Additionally, men with community-acquired pneumonia (CAP) had worse outcomes, demonstrating a 30% higher risk of mortality compared to women [15]. The higher crude and age-adjusted mortality rates observed in males may reflect a combination of biological susceptibility, behavioral risk factors, and disparities in healthcare access. Risk factors such as higher rates of smoking, chronic obstructive pulmonary disease (COPD), and occupational exposures to respiratory hazards may contribute to the increased male susceptibility to pneumonia-related complications. Marini et al. reported that male sex is an independent risk factor for pneumonia, leading to increased 90-day mortality. Implementing sex-specific preventive strategies may help reduce these complications and improve pneumonia outcomes [16]. Conversely, females show relatively lower mortality rates, potentially linked to differences in immune response and healthcare-seeking behaviors. These findings emphasize the importance of targeted public health interventions, particularly for high-risk male populations.

Racial disparities in pneumonia-related mortality persist, with Non-Hispanic Black and Non-Hispanic White populations exhibiting the highest mortality rates compared to other racial groups. Hospitals serving more African American and Hispanic patients showed lower adherence to most quality indicators. Among racial/ethnic groups, Non-Hispanic Black individuals experienced the highest pneumonia-related mortality, with an AAMRR of 1.11 (95% CI: 1.10-1.11) relative to Non-Hispanic White individuals [17]. The mortality burden among Non-Hispanic Black individuals remains disproportionately high, potentially reflecting systemic healthcare inequities, socioeconomic factors, and higher prevalence rates of chronic conditions such as diabetes, hypertension, and cardiovascular diseases. Meanwhile, the Non-Hispanic White population also experiences elevated pneumonia-related mortality rates, particularly among older adults. Other racial groups, including Asian/Pacific Islanders and American Indians/Alaska Natives, show comparatively lower rates; however, localized disparities may exist within these populations. The trends indicate an urgent need for addressing racial health disparities through improved healthcare access, vaccination programs, and early pneumonia detection strategies.

The mortality trends by Hispanic origin reveal a notable difference in pneumonia-related deaths between Hispanic and Non-Hispanic populations [18]. While Hispanic individuals generally exhibit lower crude and age-adjusted mortality rates compared to their Non-Hispanic counterparts, this gap appears to have narrowed in recent years. The Hispanic mortality advantage, often attributed to the "Hispanic Paradox," suggests that despite socioeconomic challenges, Hispanic populations may benefit from stronger familial support, healthier dietary patterns, and lower rates of smoking [18]. However, the increasing trend in pneumonia-related mortality among Hispanics in the later years of the dataset warrants further investigation into evolving risk factors, healthcare access issues, and the impact of comorbidities within this group.

The longitudinal data on pneumonia-related mortality underscore significant disparities across age groups, gender, race, and Hispanic origin. While mortality rates have generally declined over the years, specific vulnerable populations continue to experience a disproportionate burden. Public health efforts should focus on tailored interventions, including improved vaccination coverage, early pneumonia detection, and enhanced access to healthcare services [19]. Health care-seeking behavior for children with pneumonia was lower among the poorest families, those with no formal education, and children living in rural areas. To improve pneumonia treatment access and align with key UN Sustainable Development Goals (SDGs), policies and strategies must focus on these underserved populations. Addressing socioeconomic determinants and healthcare disparities is essential to reducing pneumonia-related mortality and improving outcomes for at-risk groups. These findings offer critical insights for healthcare policymakers and epidemiologists in developing targeted strategies to combat pneumonia-related mortality nationwide [20].

Strengths and limitations of this study

This study provides a comprehensive analysis of pneumonia-related mortality trends over 25 years using CDC WONDER data. The large dataset enhances reliability, and age-adjusted mortality rates allow fair comparisons across demographics. Identifying disparities by gender, race, age, and ethnicity helps highlight high-risk populations. The inclusion of confidence intervals ensures statistical precision, and the study captures the impact of COVID-19 on pneumonia mortality. However, limitations exist. Death certificate data may introduce misclassification errors, and the study lacks individual-level risk factors like smoking, vaccination, and comorbidities. Missing male mortality data after 2013 limits long-term gender trend assessment. Additionally, healthcare access disparities, which may contribute to racial and ethnic differences, are not accounted for. The study identifies trends but does not establish causal relationships between interventions and mortality declines. Despite these limitations, the findings provide a valuable foundation for future research and targeted public health interventions.

## Conclusions

The analysis of pneumonia-related mortality trends from 1999 to 2023 highlights a significant decline in overall deaths and age-adjusted mortality rates, indicating progress in public health and medical interventions. Thus, the analysis has disclosed significant demographic disparities, seasonal trends, and geographic variations. Even though there has been a substantial decline in overall mortality, indicating improvements in treatment, prevention, and access to healthcare, persistent gaps have remained, especially among age and racial groups. Consequently, both males and females exhibited declining mortality trends, though males consistently had higher rates. Racial disparities persisted, with Black or African American individuals experiencing disproportionately higher mortality rates despite improvements over time. Similarly, age-based trends showed that mortality rates increased with age, with the highest burden observed in individuals aged 85 and older compared to the younger populations, regardless of the notable declines in child and infant mortality. Still, Hispanic populations consistently had lower mortality rates than non-Hispanic populations, though disparities remained. The impact of the COVID-19 pandemic was evident in the temporary increase in mortality rates in 2020. While the overall trends reflect advancements in pneumonia prevention and treatment, persistent disparities by race, gender, and ethnicity emphasize the need for targeted public health interventions to further reduce pneumonia-related mortality, particularly in vulnerable populations. Notably, though mortality rates have stabilized after the COVID-19 pandemic, the non-Hispanic population has continued to experience higher mortality rates compared to their Hispanic counterparts. The findings of this study highlight the significant progress realized in the management of pneumonia and the ongoing requirement for effective targeted intervention to tackle persistent health inequities. 

## References

[1] Jain V, Vashisht R, Yilmaz G (2025 Jan-). Pneumonia pathology. StatPearls [Internet].

[2] Sattar SBA, Nguyen AD, Sharma S. (2025 Jan-). Bacterial pneumonia. StatPearls [Internet].

[3] Torres A, Cilloniz C, Niederman MS, Menéndez R, Chalmers JD, Wunderink RG, van der Poll T (2021). Pneumonia. Nat Rev Dis Primers.

[4] Karim R, Afridi JK, Lala GE, Yar SR, Zaman MB, Afridi BK (2023). Clinical findings and radiological evaluation of WHO-defined severe pneumonia among hospitalized children. Cureus.

[5] Dion CF, Ashurst JV (2025 Jan-). Streptococcus pneumoniae. StatPearls [Internet].

[6] (2023). Pneumonia Prevention and Control | Pneumonia | CDC. Updated Oct 13.

[7] (2025). Pneumonia Facts and Statistics: What You Need to Know | VeryWell Health. Verywell Health. Published Feb.

[8] Holland E, Jabbar AB, Asghar MS, Asghar N, Mistry K, Mirza M, Tauseef A (2025). Demographic and regional trends of pneumonia mortality in the United States, 1999 to 2022. Sci Rep.

[9] (2025). About Rural Health | Rural Health | CDC. Updated.

[10] (2025). CDC WONDER. https://wonder.cdc.gov.

[11] (2025). Pneumonia - Our World in Data | Our World in Data. https://ourworldindata.org/pneumonia.

[12] Häder A, Köse-Vogel N, Schulz L (2023). Respiratory infections in the aging lung: implications for diagnosis, therapy, and prevention. Aging Dis.

[13] Ebeledike C, Ahmad T (2025 Jan-). Pediatric pneumonia. StatPearls [Internet].

[14] Bondarchuk CP, Grobman B, Mansur A, Lu CY (2025). National trends in pneumonia-related mortality in the United States, 1999-2019. Infect Dis (Lond).

[15] Corica B, Tartaglia F, D'Amico T, Romiti GF, Cangemi R (2022). Sex and gender differences in community-acquired pneumonia. Intern Emerg Med.

[16] Marini S, Morotti A, Lena UK, Goldstein JN, Greenberg SM, Rosand J, Anderson CD (2018). Men experience higher risk of pneumonia and death after intracerebral hemorrhage. Neurocrit Care.

[17] Diaz-Campbell A, Sumon M, Mehari A, Snead MB, Ramirez R, Arend E, Gillum RF (2021). Geographic heterogeneity in influenza and pneumonia mortality in Hispanic Americans. Int J Environ Res Public Health.

[18] Chen Y, Freedman ND, Rodriquez EJ (2020). Trends in premature deaths among adults in the United States and Latin America. JAMA Netw Open.

[19] Kallander K, Burgess DH, Qazi SA (2016). Early identification and treatment of pneumonia: a call to action. Lancet Glob Health.

[20] Shibre G, Zegeye B, Idriss-Wheeler D, Yaya S (2021). Trends of inequalities in care seeking behavior for under-five children with suspected pneumonia in Ethiopia: evidence from Ethiopia demographic and health surveys (2005-2016). BMC Public Health.

